# Atypical Presentation of an Osteoid Osteoma With a Multicentric Nidus

**DOI:** 10.7759/cureus.67053

**Published:** 2024-08-17

**Authors:** Sandeep Kumar Thirlapuram, Khazi Syed Asif Hussain, Pragnya Keerthisree, Sundeep Kund Reddy Aluka

**Affiliations:** 1 Department of Orthopaedics, Nizam's Institute of Medical Sciences, Hyderabad, IND

**Keywords:** surgical excision, mri, ct, multicentric nidus, osteoid osteoma

## Abstract

An osteoid osteoma is typically a benign bone tumor affecting young adult males, often presenting with nocturnal pain alleviated by nonsteroidal anti-inflammatory medications (NSAIDS). It usually manifests as a solitary nidus with surrounding sclerosis. An osteoid osteoma with a multicentric nidus, characterized by multiple nidi, is a rare variant. A 12-year-old girl presented with a one-year history of worsening, nighttime pain in her upper left leg. Plain radiographs revealed two lytic lesions with sclerosis. A computed tomography (CT) scan confirmed two well-defined sclerotic lesions with central lytic lesions. Magnetic resonance imaging (MRI) demonstrated two hypointense lesions with peripheral hyperintensity on short tau inversion recovery (STIR) sequences, suggestive of osteoid osteoma with a multicentric nidus. Differential diagnoses included osteomyelitis with Brodie's abscess, osteoblastoma, chondroblastoma, and malignant lesions. Due to the atypical presentation and lack of experience with radiofrequency ablation (RFA) for multicentric cases, surgical excision was performed. Histopathology confirmed osteoid osteoma. After rehabilitation, the patient was asymptomatic at six months with no recurrence on radiographs. This case highlights the unusual presentation of osteoid osteoma with a multicentric nidus in a young female. Radiological workup with plain films, CT, and MRI was crucial for diagnosis. While RFA is gaining popularity, surgical excision remains a valid option, especially for atypical cases.

## Introduction

An osteoid osteoma, initially described by Henry L. Jaffe [1], is a benign bone tumor accounting for approximately 12% of all benign bone tumors [2]. This tumor commonly affects young adult males and is characterized by intense nighttime pain that is often relieved by non-steroidal anti-inflammatory drugs (NSAIDS) [3]. Osteoid osteomas primarily occur in long bones, especially the femur and tibia. Typically, a single, highly vascularized nidus of osteoid tissue develops within the tumor, surrounded by dense sclerotic bone. Computed tomography (CT) imaging modality is the investigation of choice in cortical osteoid osteoma, which demonstrates a hypodense lytic lesion called a nidus, which is surrounded by a distinct, hyperdense, thickened bone margin that is clearly separated from the normal bone. A multicentric osteoid osteoma, characterized by the presence of at least two nidi within the lesion, is a rare variant of this tumor [4]. Conventional treatment modalities for osteoid osteoma involve en bloc resection or curettage of the nidus [5]. Thermal ablation of an osteoid osteoma has gained popularity since the introduction of computed tomography (CT)-guided radiofrequency ablation (RFA) in 1992 [6]. More recently, CT-guided laser ablation has emerged as an alternative therapeutic modality [7]. This report presents a case of an osteoid osteoma with a multicentric nidus managed through en bloc resection.

## Case presentation

A 12-year-old female patient presented with a one-year history of left proximal leg pain. The patient reported a gradually worsening, intermittent pain, predominantly nocturnal, localized to the medial aspect of the proximal left leg, rated as a 7 out of 10 on a visual analog scale. The pain was not referred and was not exacerbated by any specific activity. Over-the-counter analgesics provided partial relief. There was no history of trauma, and the remaining medical and physical examination findings were unremarkable. Local examination revealed diffuse tenderness over the medial aspect of the proximal left leg. A well-circumscribed, hard, smooth bony swelling was palpable beneath intact skin. No muscle atrophy was noted. The range of motion of the left knee and ankle joints was preserved, and there were no distal neurological or vascular deficits.

**Figure 1:**
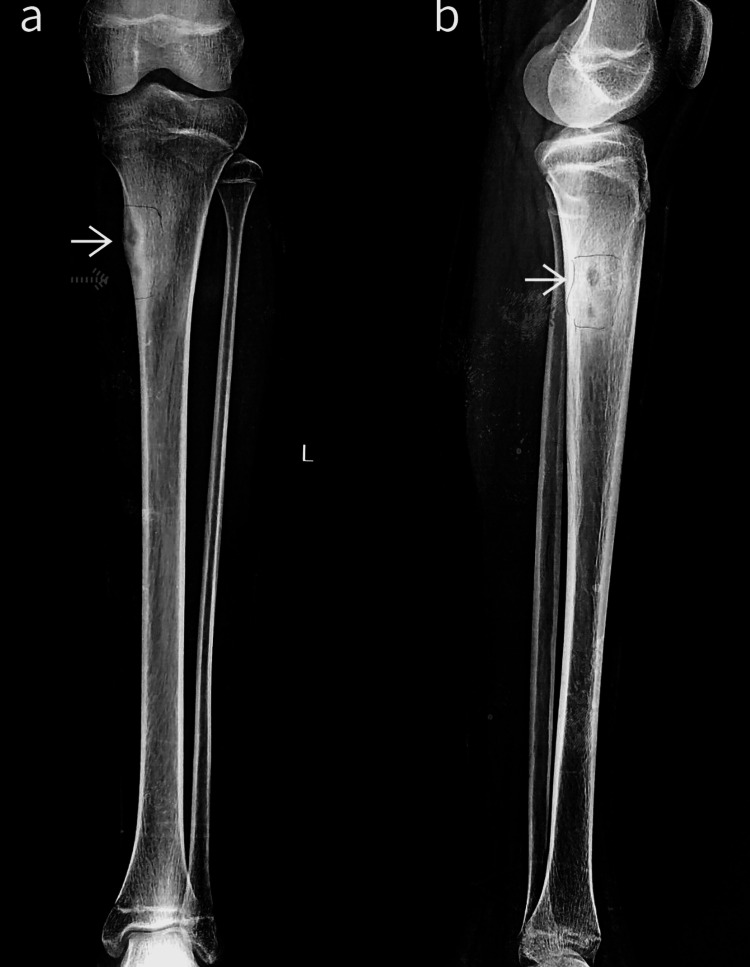
Anteroposterior (a) and lateral (b) plain radiographs of the left knee with the leg ankle Two lytic bone lesions with surrounding reactive sclerosis within the proximal meta-diaphyseal region of the medial tibial cortex (white and dotted arrows showing two nidi).

Plain radiographs demonstrated two lytic bone lesions with surrounding reactive sclerosis within the proximal meta-diaphyseal region of the tibial cortex (Figure 1).

**Figure 2:**
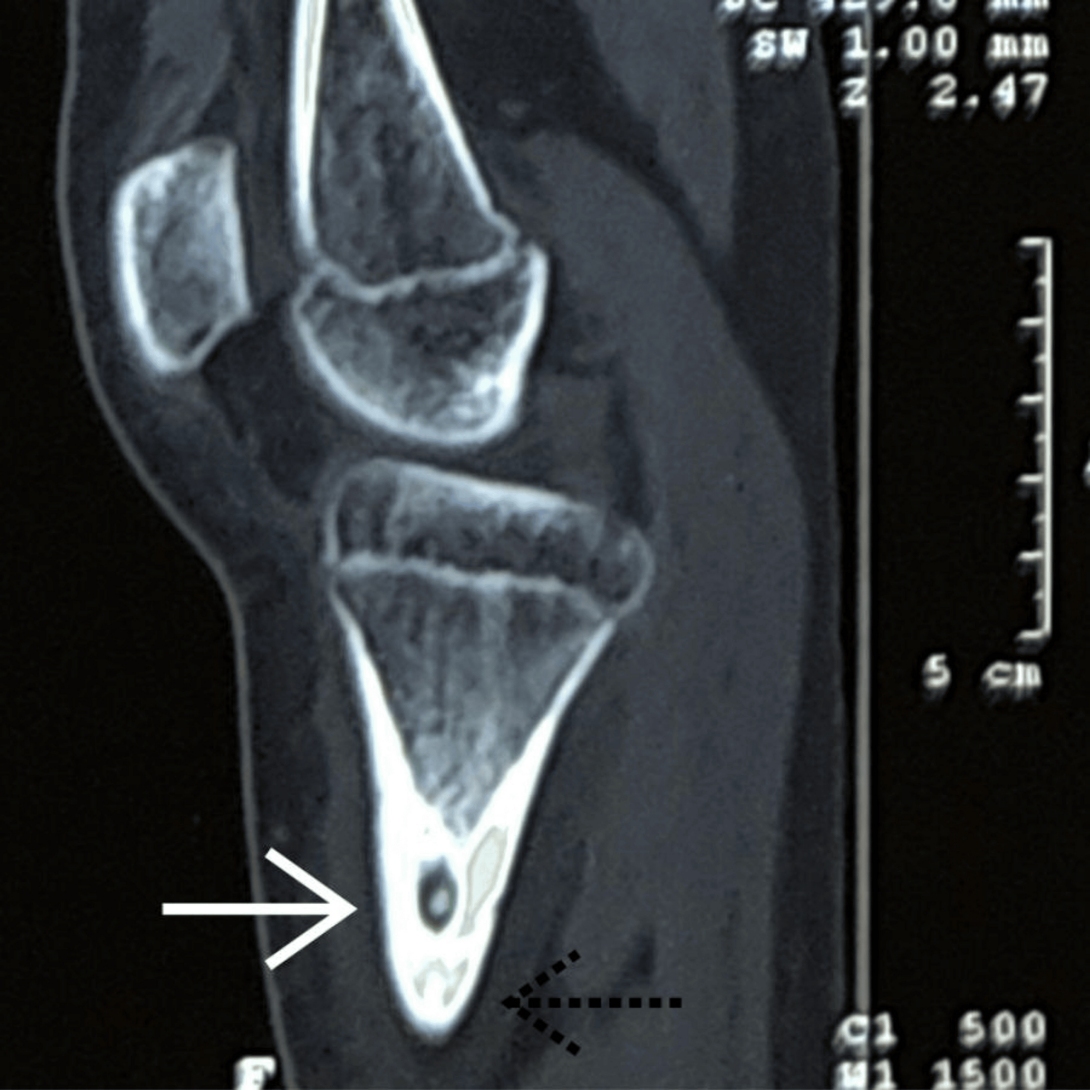
Plain CT of the left leg on the sagittal section showing an osteoid osteoma with two nidi Two well-defined sclerotic lesions with central lucency, the largest measuring 11 x 6 millimeters, at the proximal meta-diaphyseal junction of the left tibia, accompanied by adjacent cortical thickening.

CT scan revealed two well-defined sclerotic lesions with central lucencies, the largest measuring 11 x 6 millimeters, at the proximal meta-diaphyseal junction of the left tibia, accompanied by adjacent cortical thickening (Figure 2).

**Figure 3:**
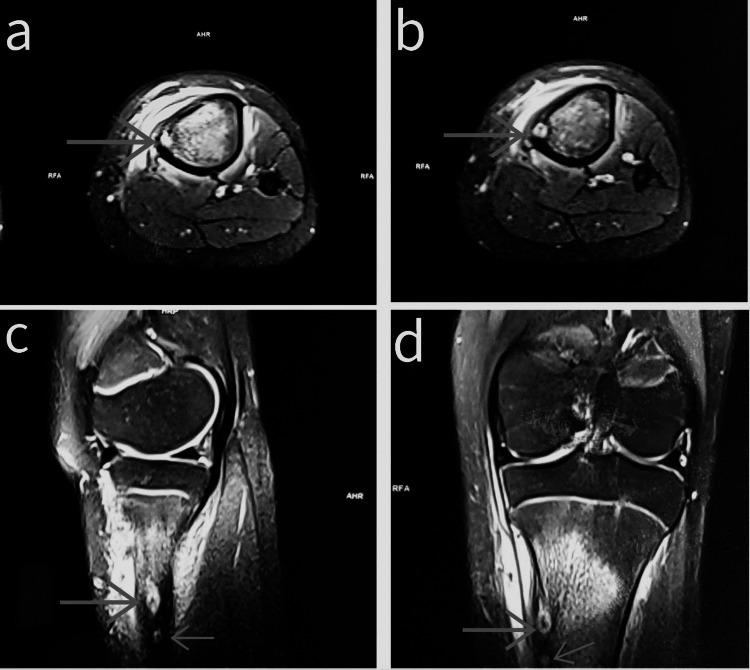
Magnetic resonance imaging short tau inversion recovery (MRI STIR) sequences axial (a,b), sagittal (c), and coronal (d) images showing an osteoid osteoma with two nidi Two small, eccentric, hypointense lesions on STIR sequences with peripheral hyperintense rims within the cortex (arrows showing two nidi).

Magnetic resonance imaging (MRI) demonstrated two small, eccentric, hypointense lesions on short tau inversion recovery (STIR) sequences with peripheral hyperintense rims within the cortex of the proximal meta-diaphyseal tibial junction, suggestive of osteoid osteoma (Figure 3). A diagnosis of osteoid osteoma with multicentric nidus was made.

**Figure 4:**
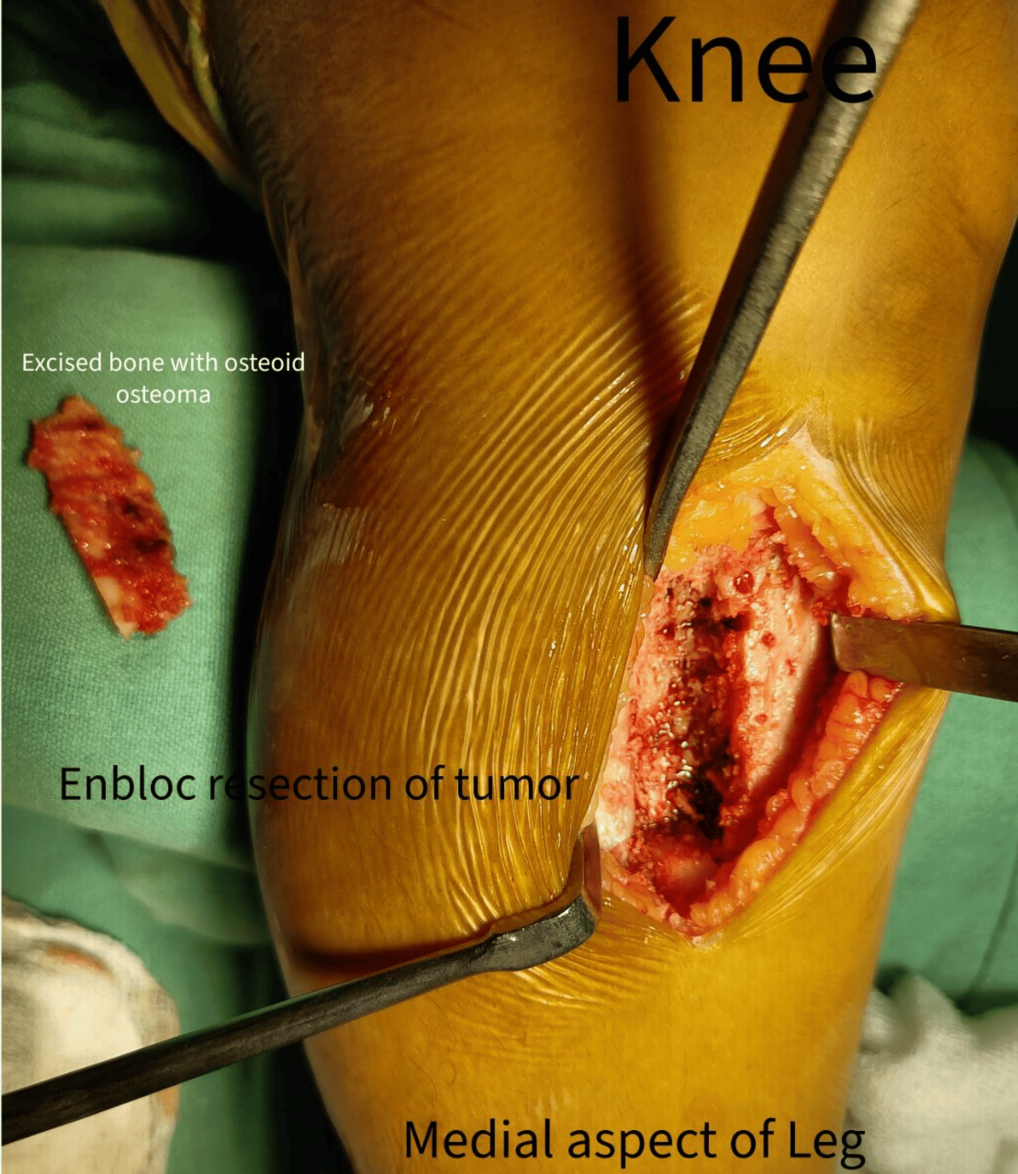
Intraoperative picture showing en bloc resection of the tumor

Radiofrequency ablation (RFA) is the preferred treatment for osteoid osteomas at our institution. However, due to a lack of experience with RFA for osteoid osteoma with multicentric nidus, surgical excision was offered and selected by the patient and their family after discussing available treatment options. The patient underwent complete excision of the lesion (Figure 4) and surrounding sclerotic bone under image guidance. The excised tissue was sent for histopathological evaluation.

**Figure 5:**
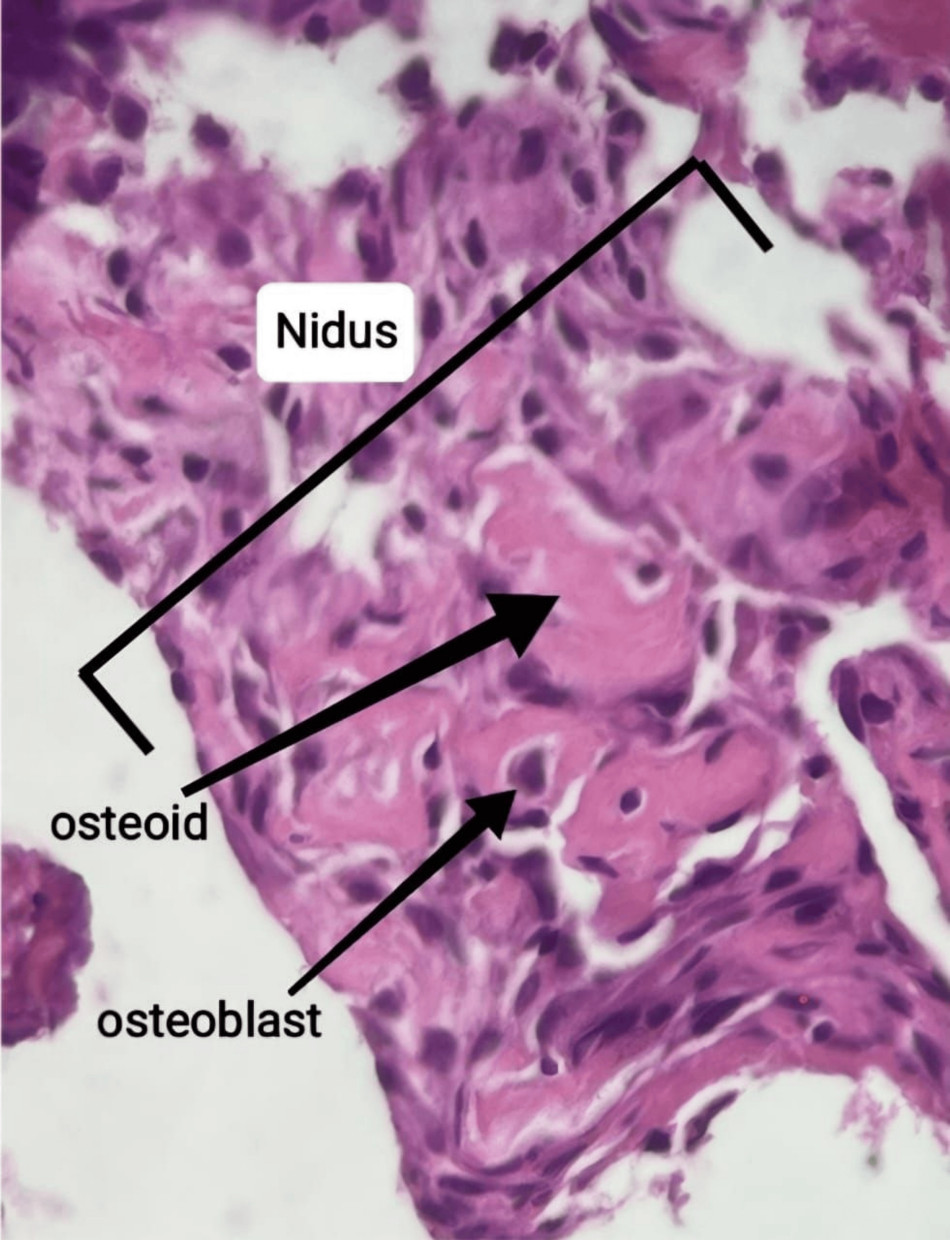
Microscopic image describing the osteoid osteoma

The histopathological report described thickened bony trabeculae and a well-defined lesion with surrounding sclerotic bone. The lesion had haphazard trabeculae of woven bone with prominent osteoblastic rimming in the background of fibrovascular stroma with bland spindle cells and scattered osteoclastic-like giant cells. These features were consistent with osteoid osteoma (Figure 5).

**Figure 6:**
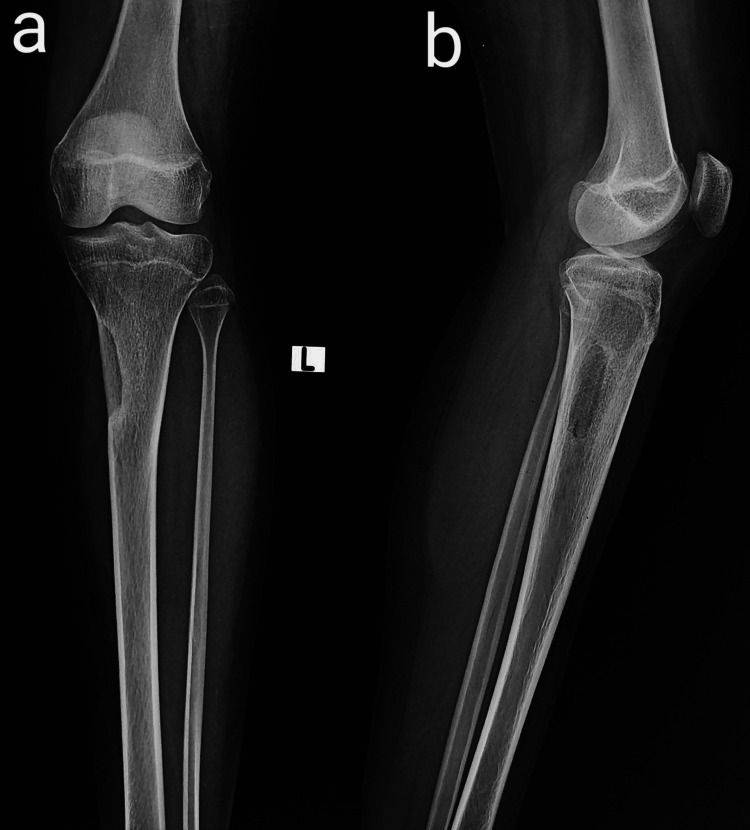
Anteroposterior (a) and lateral (b) plain radiograph of the left knee with the leg (six month postoperative follow-up) No evidence of recurrence

The patient was immobilized in a non-weight-bearing above-knee slab for four weeks, followed by a gradual progression to full weight-bearing through a period of partial weight-bearing and knee range of motion exercises as tolerated over three months. Six months post-surgery, the patient reported no symptoms, had complete knee range, no interruption in her activities of daily living, and showed no evidence of tumor recurrence (Figure 6).

## Discussion

This case report describes a unique presentation of an osteoid osteoma with a multicentric nidus in a 12-year-old female patient. Traditionally, an osteoid osteoma manifests in young adult males [8]. The patient in this case deviated from this demographic, highlighting the potential for this tumor to occur outside the expected sex and age range.

Furthermore, the presence of multiple lesions (multicentric) is uncommon, with most osteoid osteomas being solitary [9]. These atypical features posed a challenge for diagnosis. Radiological investigations are crucial for diagnosis. Plain radiographs often demonstrate a small, radiolucent nidus surrounded by sclerotic bone. In our case, plain radiographs revealed two lytic lesions with surrounding sclerosis, atypical for a solitary osteoid osteoma. CT and MRI are essential for confirming the diagnosis and delineating the extent of the lesion. Our patient's CT scan showed two well-defined sclerotic lesions with radiolucent centers, while MRI revealed two small eccentric STIR hypointense lesions with peripheral hyperintense rims, consistent with osteoid osteoma with multicentric nidus.

The differential diagnosis for an osteoid osteoma with a multicentric nidus includes osteomyelitis with Brodie’s abscess, osteoblastoma, and chondroblastoma. Malignant lesions such as osteosarcoma and Ewing sarcoma should also be considered. Only around 25 cases of osteoid osteoma with multicentric nidus have been reported in the literature [10]. In our case, the patient was initially referred to our institution with a presumptive diagnosis of osteomyelitis with Brodie's abscess. A comprehensive evaluation, including a meticulous clinical assessment, radiological imaging, and definitive histopathological analysis, led to a revised diagnosis of a multicentric osteoid osteoma.

Conventional treatment for osteoid osteomas involves en bloc resection or curettage of the nidus [11]. However, with the advent of minimally invasive techniques like CT-guided RFA and laser ablation, these procedures have become increasingly favored due to their smaller incision size, shorter recovery times, and reduced complication rates compared to traditional surgery [6,7]. 

In this particular case, due to the atypical presentation of the osteoid osteoma with a multicentric nidus, the lack of experience with RFA for such cases at our institution, and likely a desire for definitive treatment, surgical excision was the chosen modality. The patient underwent successful en bloc resection of the lesions, followed by a rehabilitation program that included non-weight bearing for four weeks, progressing to full weight bearing over three months. At the six-month follow-up, the patient was asymptomatic with full range of motion in the knee and no signs of recurrence on radiographs.

## Conclusions

This case highlights the importance of recognizing atypical radiological features of the commonly benign osteoid osteoma. While typically solitary, this tumor can rarely present with multiple nidi, creating a "string-of-beads" appearance in various imaging studies. Differential diagnoses for well-defined lytic bone lesions with associated sclerosis or sequestrum should include osteoid osteoma, despite its benign nature, due to the potential imaging overlap with Brodie's abscess.
